# Crystal structure of (piperidine-1-carbo­di­thio­ato-κ^2^ *S*,*S*)[2-(pyridin-2-yl)phenyl-κ^2^ *C* ^1^,*N*]palladium(II)

**DOI:** 10.1107/S2056989015015005

**Published:** 2015-08-15

**Authors:** Mikhail Kondrashov, André Fleckhaus, Roman Gritcenko, Ola F. Wendt

**Affiliations:** a Centre for Analysis and Synthesis, Department of Chemistry, Lund University, PO Box 124, S-221 00 Lund, Sweden

**Keywords:** crystal structure, palladium, phenyl­pyridine, di­thio­carbamate

## Abstract

The title compound, [Pd(C_11_H_8_N)(C_6_H_10_NS_2_)], crystallizes with three similar and discrete mol­ecules in the asymmetric unit. The CNS_2_ donor set defines a distorted square-planar geometry around the Pd^II^ atom, with very small deviations from planarity. The bidentate nature of the ligands gives fairly large deviations from the ideal 90° angles; the C—Pd—N angles are all around 81° and the S—Pd—S angles are around 75°. Mol­ecules pack *via* dispersion inter­actions.

## Related literature   

For structures of phenyl­pyridine with palladium, see: Nasielski *et al.* (2010 ▸). For a hexa­thia­adamantane structure with an S—Pd—S moiety, see: Pickardt & Rautenberg (1986 ▸). For examples of dinuclear palladium(II) complexes relevant to possible C—H activation, see: Powers *et al.* (2009 ▸, 2010 ▸). For the preparation of the di­thio­carbamic acid, see: Kiss (2007 ▸).
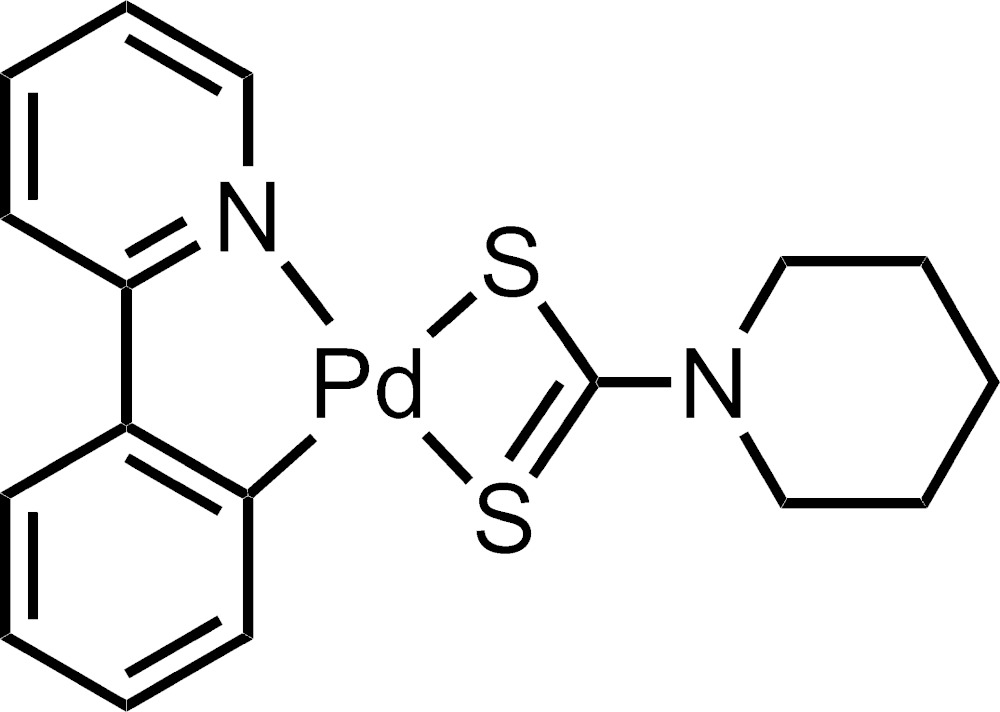


## Experimental   

### Crystal data  

[Pd(C_11_H_8_N)(C_6_H_10_NS_2_)]
*M* *_r_* = 420.85
Monoclinic, 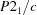

*a* = 24.0780 (9) Å
*b* = 8.5585 (2) Å
*c* = 26.6841 (10) Å
β = 113.514 (4)°
*V* = 5042.2 (3) Å^3^
*Z* = 12
Mo *K*α radiation
μ = 1.35 mm^−1^
*T* = 293 K
0.25 × 0.15 × 0.03 mm

### Data collection  

Agilent Xcalibur Sapphire3 diffractometer
Absorption correction: multi-scan (*CrysAlis PRO*; Agilent, 2011 ▸) *T*_min_ = 0.604, *T*_max_ = 1.000
58403 measured reflections
12332 independent reflections
8246 reflections with *I* > 2σ(*I*)
*R*_int_ = 0.057

### Refinement  

*R*[*F* ^2^ > 2σ(*F* ^2^)] = 0.052
*wR*(*F* ^2^) = 0.096
*S* = 1.08
12332 reflections
595 parameters
H-atom parameters constrained
Δρ_max_ = 0.78 e Å^−3^
Δρ_min_ = −0.69 e Å^−3^

### 

Data collection: *CrysAlis PRO* (Agilent, 2011 ▸); cell refinement: *CrysAlis PRO*; data reduction: *CrysAlis PRO*; program(s) used to solve structure: *SIR92* (Altomare *et al.*, 1994 ▸); program(s) used to refine structure: *SHELXL2014* (Sheldrick, 2008 ▸); molecular graphics: *CrystalMaker* (CrystalMaker, 2011 ▸); software used to prepare material for publication: *SHELXL2014* (Sheldrick, 2008 ▸).

## Supplementary Material

Crystal structure: contains datablock(s) I. DOI: 10.1107/S2056989015015005/tk5379sup1.cif

Structure factors: contains datablock(s) I. DOI: 10.1107/S2056989015015005/tk5379Isup2.hkl

**Fig. 1.**
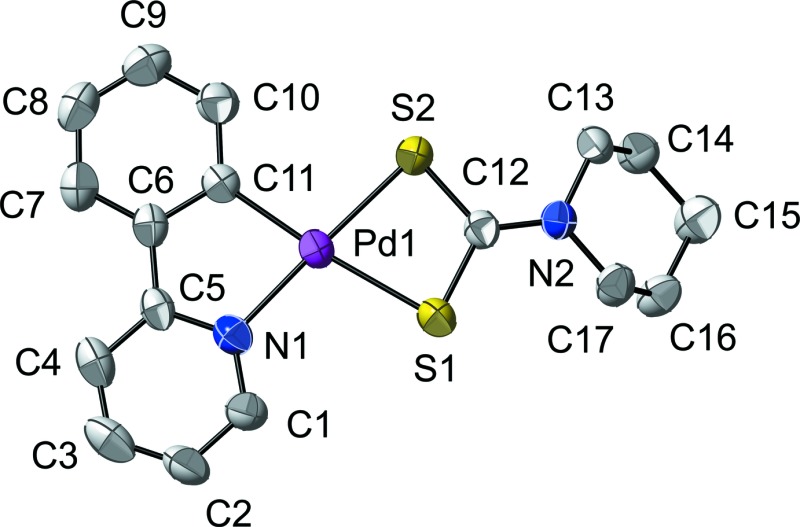
The molecular structure of one of the molecules in the asymmetric unit with atom labels and 50% probability displacement ellipsoids. H-atoms are omitted for clarity.

. DOI: 10.1107/S2056989015015005/tk5379fig1.tif
The mol­ecular structure of one of the mol­ecules in the asymmetric unit with atom labels and 50% probability displacement ellipsoids. H-atoms are omitted for clarity.

CCDC reference: 1418104

Additional supporting information:  crystallographic information; 3D view; checkCIF report

## supplementary crystallographic information

### S1. Structural commentary

We were inter­ested in synthesizing dinuclear Pd^II^ complexes because of their possible involvement in C—H activation (Powers *et al.*, 2009; Powers *et al.*, 2010). The title compound was synthesized by a ligand exchange from a corresponding acetate-bridged dimer (Powers *et al.* (2009) and *N*-piperidine­dithio­carbamic acid (Kiss, 2007). However, despite the isoelectronic structure and similar geometry of acetate and di­thio­carbamate, the product formed was found to have a monomeric structure. The likely explanation for this difference is that the larger atomic radius and longer bonds of sulfur decrease the strain in the four-membered ring that is formed in a monomeric structure. The asymmetric unit contains three discrete molecules and there is no indication of any strong inter­molecular forces; packing is by dispersion.The natural bite angle of the ligands make the angles smaller than 90° and there is good agreement with other phenyl­pyridine palladium(II) complexes (Nasielski *et al.* 2010), and also with an example of a hexa­thia­adamantane structure displaying the same S–Pd–S moiety as seen here (Pickardt and Rautenberg, 1986). Bond distances are unremarkable and the higher trans influence of the σ-C compared to the nitro­gen is clearly seen in the Pd–S bond which is approximately 0.1 Å longer *trans* to carbon.

### S2. Synthesis and crystallization

In air, [(phpy)PdOAc]_2_ (20 mg, 0.034 mmol, 1 equiv) was added to a solution of N-piperidine­dithio­carbamic acid (11 mg, 0.068 mmol, 2 equiv) in MeCN (10 mL). The resulting solution was stirred overnight at 40°C, and then it was cooled to RT. The reaction mixture was concentrated to ~1mL and a mixture of Et_2_O/pentane (3:1, 5 mL) was added. The bright-yellow precipitate formed was filtered off and washed with mixture of Et_2_O/pentane (3:1, 3x2 mL) and dried. 23 mg (80%) of the title compound was obtained. X-ray quality crystals were obtained by recrystallization from a CH_2_Cl_2_/MeCN solution.

^1^H NMR (500 MHz, CD_2_Cl_2_): d 8.38 (d, *J* = 5 Hz, 1H, H_1_), 7.86 (m, 1H, H_3_), 7.80 (d, *J* = 8 Hz, 1H, H_4_), 7.59 (dd, *J* = 7, 1 Hz, 1H, H_7_), 7.20 – 7.00 (m, 4H, H_2,8-10_), 4.01 (dd, *J* = 11, 5 Hz, 4H, H_13+17_), 1.80 – 1.64 (m, 6H, H_14-16_)

### S3. Refinement

The H atoms were positioned geometrically and treated as riding on their parent atoms with C–H distances of 0.93–0.97 Å, and with *U*_iso_(H) = 1.2 *U*_eq_.

### Crystal data

**Table:**

[Pd(C_11_H_8_N)(C_6_H_10_NS_2_)]	*F*(000) = 2544
*M**_r_* = 420.85	*D*_x_ = 1.663 Mg m^−^^3^
Monoclinic, *P*2_1_/*c*	Mo *K*α radiation, λ = 0.71073 Å
*a* = 24.0780 (9) Å	Cell parameters from 8955 reflections
*b* = 8.5585 (2) Å	θ = 2.5–29.0°
*c* = 26.6841 (10) Å	µ = 1.35 mm^−^^1^
β = 113.514 (4)°	*T* = 293 K
*V* = 5042.2 (3) Å^3^	Plate, yellow
*Z* = 12	0.25 × 0.15 × 0.03 mm

### Data collection

**Table:**

Agilent Xcalibur Sapphire3 diffractometer	12332 independent reflections
Radiation source: Enhance (Mo) X-ray Source	8246 reflections with *I* > 2σ(*I*)
Graphite monochromator	*R*_int_ = 0.057
Detector resolution: 16.1829 pixels mm^-1^	θ_max_ = 29.1°, θ_min_ = 2.5°
ω scans	*h* = −32→31
Absorption correction: multi-scan (*CrysAlis PRO*; Agilent, 2011)	*k* = −11→11
*T*_min_ = 0.604, *T*_max_ = 1.000	*l* = −34→35
58403 measured reflections	

### Refinement

**Table:**

Refinement on *F*^2^	0 restraints
Least-squares matrix: full	Hydrogen site location: inferred from neighbouring sites
*R*[*F*^2^ > 2σ(*F*^2^)] = 0.052	H-atom parameters constrained
*wR*(*F*^2^) = 0.096	*w* = 1/[σ^2^(*F*_o_^2^) + (0.0258*P*)^2^ + 4.0221*P*] where *P* = (*F*_o_^2^ + 2*F*_c_^2^)/3
*S* = 1.08	(Δ/σ)_max_ = 0.001
12332 reflections	Δρ_max_ = 0.78 e Å^−^^3^
595 parameters	Δρ_min_ = −0.69 e Å^−^^3^

### Special details

**Table:**

Geometry. All e.s.d.'s (except the e.s.d. in the dihedral angle between two l.s. planes) are estimated using the full covariance matrix. The cell e.s.d.'s are taken into account individually in the estimation of e.s.d.'s in distances, angles and torsion angles; correlations between e.s.d.'s in cell parameters are only used when they are defined by crystal symmetry. An approximate (isotropic) treatment of cell e.s.d.'s is used for estimating e.s.d.'s involving l.s. planes.

### Fractional atomic coordinates and isotropic or equivalent isotropic displacement parameters (Å^2^)

**Table:**

	*x*	*y*	*z*	*U*_iso_*/*U*_eq_	
C1	0.47872 (19)	0.9806 (5)	0.59090 (18)	0.0540 (11)	
H1	0.4729	0.8838	0.6039	0.065*	
C2	0.5189 (2)	1.0838 (6)	0.6266 (2)	0.0637 (13)	
H2	0.5398	1.0577	0.6632	0.076*	
C3	0.5275 (2)	1.2265 (6)	0.6070 (2)	0.0676 (14)	
H3	0.5550	1.2974	0.6303	0.081*	
C4	0.4954 (2)	1.2637 (5)	0.5531 (2)	0.0590 (13)	
H4	0.5008	1.3610	0.5401	0.071*	
C5	0.45500 (17)	1.1572 (4)	0.51770 (18)	0.0429 (10)	
C6	0.41895 (17)	1.1804 (4)	0.45974 (18)	0.0425 (10)	
C11	0.38240 (17)	1.0550 (4)	0.43218 (16)	0.0400 (9)	
C12	0.33523 (17)	0.5711 (5)	0.47067 (16)	0.0421 (9)	
C13	0.25652 (19)	0.3891 (5)	0.41511 (17)	0.0503 (11)	
H13A	0.2655	0.2870	0.4044	0.060*	
H13B	0.2527	0.4626	0.3863	0.060*	
C14	0.19769 (18)	0.3824 (5)	0.42284 (18)	0.0536 (11)	
H14A	0.1658	0.3430	0.3898	0.064*	
H14B	0.1865	0.4867	0.4296	0.064*	
C15	0.2039 (2)	0.2771 (6)	0.47048 (19)	0.0656 (14)	
H15A	0.1670	0.2814	0.4768	0.079*	
H15B	0.2097	0.1701	0.4616	0.079*	
C16	0.25689 (19)	0.3259 (5)	0.52196 (18)	0.0550 (12)	
H16A	0.2486	0.4275	0.5336	0.066*	
H16B	0.2618	0.2515	0.5509	0.066*	
C17	0.31480 (18)	0.3338 (5)	0.51270 (18)	0.0515 (11)	
H17A	0.3474	0.3730	0.5453	0.062*	
H17B	0.3258	0.2301	0.5051	0.062*	
N1	0.44742 (14)	1.0153 (4)	0.53759 (14)	0.0460 (8)	
N2	0.30608 (14)	0.4380 (4)	0.46629 (13)	0.0445 (8)	
S1	0.39245 (5)	0.63462 (13)	0.53043 (5)	0.0510 (3)	
S2	0.31981 (5)	0.70232 (13)	0.41777 (4)	0.0500 (3)	
Pd1	0.38867 (2)	0.86906 (3)	0.48024 (2)	0.04174 (9)	
C42	−0.1449 (3)	0.3674 (7)	0.1333 (2)	0.0747 (15)	
H42	−0.1863	0.3804	0.1138	0.090*	
C43	−0.1223 (3)	0.2369 (7)	0.1647 (2)	0.0768 (16)	
H43	−0.1487	0.1610	0.1674	0.092*	
C44	−0.0605 (3)	0.2182 (6)	0.1925 (2)	0.0716 (15)	
H44	−0.0453	0.1289	0.2134	0.086*	
C45	−0.0210 (2)	0.3326 (5)	0.18941 (18)	0.0532 (12)	
C46	0.0450 (2)	0.3269 (5)	0.21499 (18)	0.0535 (11)	
C47	0.0774 (3)	0.2004 (6)	0.2458 (2)	0.0753 (16)	
H47	0.0571	0.1136	0.2510	0.090*	
C48	0.1401 (3)	0.2049 (7)	0.2685 (2)	0.0793 (17)	
H48	0.1618	0.1208	0.2891	0.095*	
C49	0.1703 (3)	0.3323 (6)	0.2609 (2)	0.0709 (14)	
H49	0.2124	0.3349	0.2766	0.085*	
C50	0.1381 (2)	0.4575 (5)	0.22969 (18)	0.0571 (12)	
H50	0.1589	0.5429	0.2244	0.068*	
C51	0.0755 (2)	0.4574 (5)	0.20639 (17)	0.0473 (10)	
C52	0.03404 (18)	0.9253 (5)	0.12234 (16)	0.0453 (10)	
C53	0.10411 (19)	1.1262 (5)	0.11693 (18)	0.0578 (12)	
H53A	0.1342	1.0456	0.1335	0.069*	
H53B	0.1119	1.2115	0.1428	0.069*	
C54	0.1088 (2)	1.1846 (6)	0.06519 (19)	0.0626 (13)	
H54A	0.1483	1.2317	0.0743	0.075*	
H54B	0.1052	1.0970	0.0410	0.075*	
C55	0.0602 (2)	1.3033 (6)	0.0360 (2)	0.0693 (14)	
H55A	0.0657	1.3951	0.0588	0.083*	
H55B	0.0633	1.3352	0.0023	0.083*	
C56	−0.0014 (2)	1.2336 (5)	0.02334 (19)	0.0635 (13)	
H56A	−0.0086	1.1498	−0.0030	0.076*	
H56B	−0.0322	1.3129	0.0070	0.076*	
C57	−0.0066 (2)	1.1706 (5)	0.07404 (19)	0.0575 (12)	
H57A	−0.0061	1.2570	0.0978	0.069*	
H57B	−0.0450	1.1167	0.0638	0.069*	
N5	−0.04432 (17)	0.4636 (4)	0.15885 (15)	0.0528 (9)	
N6	0.04299 (15)	1.0624 (4)	0.10399 (14)	0.0467 (8)	
S5	0.09201 (5)	0.80207 (14)	0.16127 (5)	0.0576 (3)	
S6	−0.03610 (5)	0.84672 (14)	0.10983 (5)	0.0585 (3)	
Pd3	0.02011 (2)	0.62500 (4)	0.16033 (2)	0.04749 (10)	
C7	0.4200 (2)	1.3170 (5)	0.4314 (2)	0.0562 (12)	
H7	0.4442	1.4006	0.4497	0.067*	
C8	0.3848 (2)	1.3272 (5)	0.3759 (2)	0.0625 (13)	
H8	0.3849	1.4186	0.3571	0.075*	
C9	0.3500 (2)	1.2038 (6)	0.3487 (2)	0.0606 (13)	
H9	0.3272	1.2102	0.3112	0.073*	
C10	0.3488 (2)	1.0690 (5)	0.37684 (18)	0.0545 (11)	
H10	0.3248	0.9857	0.3579	0.065*	
C21	0.4498 (2)	0.0167 (6)	0.1723 (2)	0.0633 (13)	
H21	0.4504	0.1109	0.1551	0.076*	
C22	0.4939 (2)	−0.0922 (7)	0.1785 (2)	0.0749 (15)	
H22	0.5235	−0.0731	0.1652	0.090*	
C23	0.4934 (3)	−0.2304 (7)	0.2049 (2)	0.0777 (16)	
H23	0.5239	−0.3039	0.2109	0.093*	
C24	0.4481 (2)	−0.2592 (6)	0.22206 (19)	0.0699 (15)	
H24	0.4470	−0.3541	0.2386	0.084*	
C25	0.4037 (2)	−0.1475 (5)	0.21495 (17)	0.0544 (12)	
C26	0.3526 (2)	−0.1629 (5)	0.23108 (17)	0.0529 (12)	
C27	0.3415 (2)	−0.2961 (6)	0.25529 (19)	0.0652 (14)	
H27	0.3670	−0.3822	0.2620	0.078*	
C28	0.2925 (3)	−0.2998 (6)	0.26931 (19)	0.0723 (16)	
H28	0.2856	−0.3882	0.2863	0.087*	
C29	0.2534 (2)	−0.1751 (6)	0.25874 (19)	0.0689 (14)	
H29	0.2200	−0.1791	0.2680	0.083*	
C30	0.2648 (2)	−0.0418 (5)	0.23374 (17)	0.0584 (12)	
H30	0.2385	0.0428	0.2264	0.070*	
C31	0.3136 (2)	−0.0332 (5)	0.21989 (16)	0.0488 (11)	
C32	0.29763 (19)	0.4484 (5)	0.14680 (16)	0.0474 (10)	
C33	0.2954 (2)	0.6764 (5)	0.09022 (18)	0.0541 (11)	
H33A	0.3330	0.6322	0.0912	0.065*	
H33B	0.3037	0.7812	0.1052	0.065*	
C34	0.2494 (2)	0.6829 (6)	0.03194 (18)	0.0614 (13)	
H34A	0.2450	0.5797	0.0158	0.074*	
H34B	0.2637	0.7530	0.0111	0.074*	
C35	0.1882 (2)	0.7388 (6)	0.0286 (2)	0.0720 (15)	
H35A	0.1910	0.8477	0.0395	0.086*	
H35B	0.1587	0.7308	−0.0088	0.086*	
C36	0.1675 (2)	0.6421 (6)	0.06549 (18)	0.0614 (13)	
H36A	0.1306	0.6868	0.0658	0.074*	
H36B	0.1584	0.5368	0.0511	0.074*	
C37	0.2145 (2)	0.6357 (5)	0.12260 (18)	0.0584 (12)	
H37A	0.2199	0.7390	0.1388	0.070*	
H37B	0.2011	0.5662	0.1443	0.070*	
C41	−0.1044 (2)	0.4787 (6)	0.1314 (2)	0.0636 (13)	
H41	−0.1193	0.5677	0.1102	0.076*	
N3	0.40623 (16)	−0.0083 (4)	0.19014 (14)	0.0527 (9)	
N4	0.27226 (16)	0.5801 (4)	0.12316 (14)	0.0499 (9)	
S3	0.36489 (5)	0.37714 (14)	0.14774 (5)	0.0570 (3)	
S4	0.26594 (6)	0.32525 (13)	0.17964 (5)	0.0571 (3)	
Pd2	0.34020 (2)	0.14795 (4)	0.18645 (2)	0.04822 (10)	

### Atomic displacement parameters (Å^2^)

**Table:**

	*U*^11^	*U*^22^	*U*^33^	*U*^12^	*U*^13^	*U*^23^
C1	0.057 (3)	0.058 (3)	0.044 (3)	−0.006 (2)	0.018 (2)	−0.005 (2)
C2	0.059 (3)	0.078 (3)	0.049 (3)	−0.005 (3)	0.016 (2)	−0.020 (3)
C3	0.051 (3)	0.074 (4)	0.069 (4)	−0.015 (3)	0.016 (3)	−0.030 (3)
C4	0.053 (3)	0.047 (3)	0.078 (4)	−0.011 (2)	0.027 (3)	−0.015 (3)
C5	0.037 (2)	0.040 (2)	0.056 (3)	−0.0019 (19)	0.023 (2)	−0.008 (2)
C6	0.038 (2)	0.038 (2)	0.057 (3)	0.0010 (18)	0.026 (2)	−0.003 (2)
C11	0.037 (2)	0.040 (2)	0.045 (3)	0.0036 (18)	0.0189 (19)	−0.0031 (19)
C12	0.040 (2)	0.044 (2)	0.046 (2)	−0.0016 (19)	0.0220 (19)	−0.0047 (19)
C13	0.056 (3)	0.049 (2)	0.045 (3)	−0.016 (2)	0.020 (2)	−0.010 (2)
C14	0.039 (2)	0.067 (3)	0.052 (3)	−0.007 (2)	0.015 (2)	0.005 (2)
C15	0.046 (3)	0.082 (3)	0.069 (3)	−0.009 (3)	0.022 (2)	0.014 (3)
C16	0.056 (3)	0.056 (3)	0.055 (3)	0.008 (2)	0.025 (2)	0.016 (2)
C17	0.047 (3)	0.049 (2)	0.056 (3)	−0.002 (2)	0.017 (2)	0.010 (2)
N1	0.0406 (19)	0.050 (2)	0.049 (2)	−0.0007 (16)	0.0192 (17)	−0.0078 (17)
N2	0.044 (2)	0.0458 (19)	0.045 (2)	−0.0068 (17)	0.0192 (16)	0.0000 (17)
S1	0.0469 (6)	0.0522 (6)	0.0465 (6)	−0.0083 (5)	0.0108 (5)	−0.0018 (5)
S2	0.0547 (7)	0.0481 (6)	0.0446 (6)	−0.0095 (5)	0.0170 (5)	0.0023 (5)
Pd1	0.04080 (18)	0.03782 (16)	0.04519 (19)	−0.00455 (14)	0.01567 (14)	−0.00407 (15)
C42	0.073 (4)	0.083 (4)	0.074 (4)	−0.025 (3)	0.036 (3)	−0.020 (3)
C43	0.095 (5)	0.073 (4)	0.077 (4)	−0.036 (3)	0.050 (4)	−0.017 (3)
C44	0.101 (5)	0.063 (3)	0.060 (3)	−0.019 (3)	0.042 (3)	0.002 (3)
C45	0.083 (4)	0.048 (3)	0.042 (3)	−0.007 (2)	0.039 (3)	−0.005 (2)
C46	0.075 (3)	0.053 (3)	0.041 (3)	0.007 (2)	0.032 (2)	0.003 (2)
C47	0.106 (5)	0.068 (3)	0.066 (4)	0.008 (3)	0.049 (3)	0.020 (3)
C48	0.102 (5)	0.083 (4)	0.057 (3)	0.030 (4)	0.036 (3)	0.026 (3)
C49	0.079 (4)	0.081 (4)	0.056 (3)	0.014 (3)	0.031 (3)	0.004 (3)
C50	0.067 (3)	0.056 (3)	0.052 (3)	0.004 (3)	0.028 (2)	−0.001 (2)
C51	0.062 (3)	0.043 (2)	0.044 (3)	−0.002 (2)	0.029 (2)	−0.006 (2)
C52	0.047 (2)	0.052 (2)	0.042 (2)	0.001 (2)	0.024 (2)	0.000 (2)
C53	0.049 (3)	0.061 (3)	0.059 (3)	−0.011 (2)	0.016 (2)	0.002 (2)
C54	0.058 (3)	0.072 (3)	0.062 (3)	−0.022 (3)	0.029 (3)	−0.003 (3)
C55	0.084 (4)	0.066 (3)	0.058 (3)	−0.018 (3)	0.028 (3)	0.013 (3)
C56	0.071 (3)	0.055 (3)	0.057 (3)	0.001 (3)	0.018 (3)	0.012 (2)
C57	0.051 (3)	0.054 (3)	0.066 (3)	0.004 (2)	0.022 (2)	0.008 (2)
N5	0.066 (3)	0.050 (2)	0.052 (2)	−0.006 (2)	0.033 (2)	−0.0086 (19)
N6	0.040 (2)	0.050 (2)	0.050 (2)	−0.0003 (17)	0.0174 (16)	0.0075 (18)
S5	0.0478 (7)	0.0572 (7)	0.0656 (8)	0.0043 (6)	0.0202 (6)	0.0132 (6)
S6	0.0443 (6)	0.0604 (7)	0.0729 (8)	−0.0049 (6)	0.0255 (6)	0.0081 (6)
Pd3	0.0554 (2)	0.04311 (18)	0.0490 (2)	−0.00138 (16)	0.02612 (16)	−0.00015 (16)
C7	0.053 (3)	0.045 (2)	0.073 (4)	−0.004 (2)	0.028 (3)	0.001 (2)
C8	0.071 (3)	0.052 (3)	0.073 (4)	0.010 (3)	0.039 (3)	0.017 (3)
C9	0.068 (3)	0.063 (3)	0.054 (3)	0.014 (3)	0.027 (3)	0.009 (3)
C10	0.060 (3)	0.048 (2)	0.052 (3)	0.005 (2)	0.019 (2)	−0.002 (2)
C21	0.052 (3)	0.065 (3)	0.065 (3)	−0.006 (3)	0.015 (3)	−0.009 (3)
C22	0.056 (3)	0.091 (4)	0.068 (4)	0.003 (3)	0.015 (3)	−0.020 (3)
C23	0.069 (4)	0.080 (4)	0.066 (4)	0.024 (3)	0.007 (3)	−0.013 (3)
C24	0.077 (4)	0.057 (3)	0.051 (3)	0.013 (3)	0.000 (3)	0.001 (2)
C25	0.059 (3)	0.047 (2)	0.037 (2)	−0.001 (2)	−0.003 (2)	−0.003 (2)
C26	0.058 (3)	0.052 (3)	0.032 (2)	−0.007 (2)	0.000 (2)	−0.003 (2)
C27	0.073 (4)	0.055 (3)	0.047 (3)	−0.005 (3)	0.002 (3)	0.002 (2)
C28	0.094 (4)	0.061 (3)	0.042 (3)	−0.020 (3)	0.006 (3)	0.004 (2)
C29	0.075 (4)	0.079 (4)	0.047 (3)	−0.026 (3)	0.017 (3)	−0.008 (3)
C30	0.063 (3)	0.059 (3)	0.043 (3)	−0.011 (2)	0.010 (2)	−0.008 (2)
C31	0.049 (3)	0.051 (3)	0.032 (2)	−0.006 (2)	0.002 (2)	−0.004 (2)
C32	0.052 (3)	0.049 (2)	0.035 (2)	−0.003 (2)	0.011 (2)	−0.004 (2)
C33	0.054 (3)	0.051 (3)	0.056 (3)	−0.008 (2)	0.021 (2)	0.004 (2)
C34	0.072 (3)	0.065 (3)	0.047 (3)	−0.014 (3)	0.024 (2)	0.008 (2)
C35	0.062 (3)	0.083 (4)	0.057 (3)	−0.007 (3)	0.009 (3)	0.022 (3)
C36	0.053 (3)	0.070 (3)	0.058 (3)	−0.004 (3)	0.019 (2)	0.001 (3)
C37	0.065 (3)	0.059 (3)	0.058 (3)	0.013 (2)	0.031 (2)	0.010 (2)
C41	0.061 (3)	0.065 (3)	0.065 (3)	−0.004 (3)	0.026 (3)	−0.006 (3)
N3	0.049 (2)	0.054 (2)	0.042 (2)	−0.0057 (18)	0.0042 (18)	−0.0072 (18)
N4	0.051 (2)	0.055 (2)	0.044 (2)	0.0026 (18)	0.0195 (18)	0.0077 (18)
S3	0.0483 (7)	0.0545 (7)	0.0648 (8)	0.0017 (6)	0.0189 (6)	0.0004 (6)
S4	0.0644 (8)	0.0543 (7)	0.0540 (7)	0.0005 (6)	0.0251 (6)	0.0104 (6)
Pd2	0.0496 (2)	0.04335 (18)	0.0424 (2)	−0.00218 (16)	0.00854 (15)	−0.00136 (15)

### Geometric parameters (Å, º)

**Table:**

C1—N1	1.351 (5)	C54—H54B	0.9700
C1—C2	1.374 (6)	C55—C56	1.506 (6)
C1—H1	0.9300	C55—H55A	0.9700
C2—C3	1.377 (7)	C55—H55B	0.9700
C2—H2	0.9300	C56—C57	1.508 (6)
C3—C4	1.371 (6)	C56—H56A	0.9700
C3—H3	0.9300	C56—H56B	0.9700
C4—C5	1.392 (5)	C57—N6	1.470 (5)
C4—H4	0.9300	C57—H57A	0.9700
C5—N1	1.366 (5)	C57—H57B	0.9700
C5—C6	1.454 (6)	N5—C41	1.342 (5)
C6—C11	1.397 (5)	N5—Pd3	2.065 (4)
C6—C7	1.398 (6)	S5—Pd3	2.2935 (12)
C11—C10	1.377 (5)	S6—Pd3	2.4069 (12)
C11—Pd1	2.011 (4)	C7—C8	1.384 (6)
C12—N2	1.319 (5)	C7—H7	0.9300
C12—S2	1.725 (4)	C8—C9	1.363 (6)
C12—S1	1.727 (4)	C8—H8	0.9300
C13—N2	1.471 (5)	C9—C10	1.383 (6)
C13—C14	1.512 (5)	C9—H9	0.9300
C13—H13A	0.9700	C10—H10	0.9300
C13—H13B	0.9700	C21—N3	1.331 (6)
C14—C15	1.516 (6)	C21—C22	1.372 (6)
C14—H14A	0.9700	C21—H21	0.9300
C14—H14B	0.9700	C22—C23	1.378 (7)
C15—C16	1.513 (6)	C22—H22	0.9300
C15—H15A	0.9700	C23—C24	1.364 (7)
C15—H15B	0.9700	C23—H23	0.9300
C16—C17	1.512 (6)	C24—C25	1.390 (6)
C16—H16A	0.9700	C24—H24	0.9300
C16—H16B	0.9700	C25—N3	1.376 (5)
C17—N2	1.471 (5)	C25—C26	1.461 (6)
C17—H17A	0.9700	C26—C27	1.388 (6)
C17—H17B	0.9700	C26—C31	1.408 (6)
N1—Pd1	2.045 (3)	C27—C28	1.374 (7)
S1—Pd1	2.3939 (11)	C27—H27	0.9300
S2—Pd1	2.3149 (11)	C28—C29	1.376 (7)
C42—C43	1.373 (7)	C28—H28	0.9300
C42—C41	1.378 (6)	C29—C30	1.403 (6)
C42—H42	0.9300	C29—H29	0.9300
C43—C44	1.382 (7)	C30—C31	1.368 (6)
C43—H43	0.9300	C30—H30	0.9300
C44—C45	1.390 (6)	C31—Pd2	2.016 (4)
C44—H44	0.9300	C32—N4	1.317 (5)
C45—N5	1.368 (5)	C32—S3	1.721 (4)
C45—C46	1.459 (6)	C32—S4	1.730 (4)
C46—C47	1.395 (6)	C33—N4	1.467 (5)
C46—C51	1.405 (6)	C33—C34	1.508 (6)
C47—C48	1.384 (7)	C33—H33A	0.9700
C47—H47	0.9300	C33—H33B	0.9700
C48—C49	1.371 (7)	C34—C35	1.517 (6)
C48—H48	0.9300	C34—H34A	0.9700
C49—C50	1.388 (6)	C34—H34B	0.9700
C49—H49	0.9300	C35—C36	1.514 (6)
C50—C51	1.382 (6)	C35—H35A	0.9700
C50—H50	0.9300	C35—H35B	0.9700
C51—Pd3	2.009 (4)	C36—C37	1.494 (6)
C52—N6	1.322 (5)	C36—H36A	0.9700
C52—S6	1.722 (4)	C36—H36B	0.9700
C52—S5	1.727 (4)	C37—N4	1.464 (5)
C53—N6	1.476 (5)	C37—H37A	0.9700
C53—C54	1.515 (6)	C37—H37B	0.9700
C53—H53A	0.9700	C41—H41	0.9300
C53—H53B	0.9700	N3—Pd2	2.050 (4)
C54—C55	1.511 (6)	S3—Pd2	2.3996 (12)
C54—H54A	0.9700	S4—Pd2	2.2963 (13)
			
N1—C1—C2	122.1 (4)	C55—C56—H56A	109.3
N1—C1—H1	118.9	C57—C56—H56A	109.3
C2—C1—H1	118.9	C55—C56—H56B	109.3
C1—C2—C3	118.6 (5)	C57—C56—H56B	109.3
C1—C2—H2	120.7	H56A—C56—H56B	108.0
C3—C2—H2	120.7	N6—C57—C56	111.9 (4)
C4—C3—C2	119.8 (5)	N6—C57—H57A	109.2
C4—C3—H3	120.1	C56—C57—H57A	109.2
C2—C3—H3	120.1	N6—C57—H57B	109.2
C3—C4—C5	120.5 (4)	C56—C57—H57B	109.2
C3—C4—H4	119.7	H57A—C57—H57B	107.9
C5—C4—H4	119.7	C41—N5—C45	120.1 (4)
N1—C5—C4	119.0 (4)	C41—N5—Pd3	125.7 (3)
N1—C5—C6	114.8 (3)	C45—N5—Pd3	114.2 (3)
C4—C5—C6	126.2 (4)	C52—N6—C57	122.9 (3)
C11—C6—C7	120.1 (4)	C52—N6—C53	122.4 (4)
C11—C6—C5	116.0 (4)	C57—N6—C53	114.4 (3)
C7—C6—C5	123.9 (4)	C52—S5—Pd3	88.37 (15)
C10—C11—C6	118.4 (4)	C52—S6—Pd3	84.87 (14)
C10—C11—Pd1	128.0 (3)	C51—Pd3—N5	81.04 (16)
C6—C11—Pd1	113.6 (3)	C51—Pd3—S5	98.70 (13)
N2—C12—S2	123.9 (3)	N5—Pd3—S5	179.26 (10)
N2—C12—S1	123.7 (3)	C51—Pd3—S6	173.14 (13)
S2—C12—S1	112.5 (2)	N5—Pd3—S6	105.44 (11)
N2—C13—C14	110.0 (3)	S5—Pd3—S6	74.84 (4)
N2—C13—H13A	109.7	C8—C7—C6	119.7 (4)
C14—C13—H13A	109.7	C8—C7—H7	120.1
N2—C13—H13B	109.7	C6—C7—H7	120.1
C14—C13—H13B	109.7	C9—C8—C7	120.3 (4)
H13A—C13—H13B	108.2	C9—C8—H8	119.8
C13—C14—C15	110.6 (4)	C7—C8—H8	119.8
C13—C14—H14A	109.5	C8—C9—C10	119.9 (5)
C15—C14—H14A	109.5	C8—C9—H9	120.1
C13—C14—H14B	109.5	C10—C9—H9	120.1
C15—C14—H14B	109.5	C11—C10—C9	121.6 (4)
H14A—C14—H14B	108.1	C11—C10—H10	119.2
C16—C15—C14	111.3 (4)	C9—C10—H10	119.2
C16—C15—H15A	109.4	N3—C21—C22	122.0 (5)
C14—C15—H15A	109.4	N3—C21—H21	119.0
C16—C15—H15B	109.4	C22—C21—H21	119.0
C14—C15—H15B	109.4	C21—C22—C23	118.8 (5)
H15A—C15—H15B	108.0	C21—C22—H22	120.6
C17—C16—C15	111.2 (4)	C23—C22—H22	120.6
C17—C16—H16A	109.4	C24—C23—C22	119.8 (5)
C15—C16—H16A	109.4	C24—C23—H23	120.1
C17—C16—H16B	109.4	C22—C23—H23	120.1
C15—C16—H16B	109.4	C23—C24—C25	120.3 (5)
H16A—C16—H16B	108.0	C23—C24—H24	119.8
N2—C17—C16	109.5 (3)	C25—C24—H24	119.8
N2—C17—H17A	109.8	N3—C25—C24	118.8 (5)
C16—C17—H17A	109.8	N3—C25—C26	115.1 (4)
N2—C17—H17B	109.8	C24—C25—C26	126.1 (5)
C16—C17—H17B	109.8	C27—C26—C31	120.7 (5)
H17A—C17—H17B	108.2	C27—C26—C25	123.6 (5)
C1—N1—C5	120.0 (4)	C31—C26—C25	115.8 (4)
C1—N1—Pd1	125.6 (3)	C28—C27—C26	119.5 (5)
C5—N1—Pd1	114.4 (3)	C28—C27—H27	120.2
C12—N2—C13	122.3 (3)	C26—C27—H27	120.2
C12—N2—C17	123.8 (3)	C27—C28—C29	121.2 (5)
C13—N2—C17	113.6 (3)	C27—C28—H28	119.4
C12—S1—Pd1	84.79 (14)	C29—C28—H28	119.4
C12—S2—Pd1	87.34 (14)	C28—C29—C30	118.7 (5)
C11—Pd1—N1	81.17 (15)	C28—C29—H29	120.6
C11—Pd1—S2	100.29 (12)	C30—C29—H29	120.6
N1—Pd1—S2	177.45 (10)	C31—C30—C29	121.7 (5)
C11—Pd1—S1	175.08 (12)	C31—C30—H30	119.2
N1—Pd1—S1	103.52 (10)	C29—C30—H30	119.2
S2—Pd1—S1	75.09 (4)	C30—C31—C26	118.2 (4)
C43—C42—C41	118.2 (5)	C30—C31—Pd2	128.3 (4)
C43—C42—H42	120.9	C26—C31—Pd2	113.4 (3)
C41—C42—H42	120.9	N4—C32—S3	124.3 (3)
C42—C43—C44	120.0 (5)	N4—C32—S4	123.4 (3)
C42—C43—H43	120.0	S3—C32—S4	112.3 (2)
C44—C43—H43	120.0	N4—C33—C34	109.9 (3)
C43—C44—C45	120.1 (5)	N4—C33—H33A	109.7
C43—C44—H44	119.9	C34—C33—H33A	109.7
C45—C44—H44	119.9	N4—C33—H33B	109.7
N5—C45—C44	119.0 (5)	C34—C33—H33B	109.7
N5—C45—C46	114.7 (4)	H33A—C33—H33B	108.2
C44—C45—C46	126.3 (5)	C33—C34—C35	111.7 (4)
C47—C46—C51	120.4 (5)	C33—C34—H34A	109.3
C47—C46—C45	123.5 (5)	C35—C34—H34A	109.3
C51—C46—C45	116.1 (4)	C33—C34—H34B	109.3
C48—C47—C46	119.5 (5)	C35—C34—H34B	109.3
C48—C47—H47	120.2	H34A—C34—H34B	107.9
C46—C47—H47	120.2	C36—C35—C34	110.7 (4)
C49—C48—C47	120.6 (5)	C36—C35—H35A	109.5
C49—C48—H48	119.7	C34—C35—H35A	109.5
C47—C48—H48	119.7	C36—C35—H35B	109.5
C48—C49—C50	119.9 (5)	C34—C35—H35B	109.5
C48—C49—H49	120.0	H35A—C35—H35B	108.1
C50—C49—H49	120.0	C37—C36—C35	111.8 (4)
C51—C50—C49	121.2 (5)	C37—C36—H36A	109.3
C51—C50—H50	119.4	C35—C36—H36A	109.3
C49—C50—H50	119.4	C37—C36—H36B	109.3
C50—C51—C46	118.3 (4)	C35—C36—H36B	109.3
C50—C51—Pd3	127.8 (3)	H36A—C36—H36B	107.9
C46—C51—Pd3	113.9 (3)	N4—C37—C36	110.6 (4)
N6—C52—S6	124.5 (3)	N4—C37—H37A	109.5
N6—C52—S5	123.6 (3)	C36—C37—H37A	109.5
S6—C52—S5	111.9 (2)	N4—C37—H37B	109.5
N6—C53—C54	109.8 (4)	C36—C37—H37B	109.5
N6—C53—H53A	109.7	H37A—C37—H37B	108.1
C54—C53—H53A	109.7	N5—C41—C42	122.5 (5)
N6—C53—H53B	109.7	N5—C41—H41	118.8
C54—C53—H53B	109.7	C42—C41—H41	118.8
H53A—C53—H53B	108.2	C21—N3—C25	120.3 (4)
C55—C54—C53	111.6 (4)	C21—N3—Pd2	125.8 (3)
C55—C54—H54A	109.3	C25—N3—Pd2	113.9 (3)
C53—C54—H54A	109.3	C32—N4—C37	122.7 (4)
C55—C54—H54B	109.3	C32—N4—C33	123.6 (4)
C53—C54—H54B	109.3	C37—N4—C33	113.4 (3)
H54A—C54—H54B	108.0	C32—S3—Pd2	84.42 (15)
C56—C55—C54	109.9 (4)	C32—S4—Pd2	87.47 (15)
C56—C55—H55A	109.7	C31—Pd2—N3	81.62 (17)
C54—C55—H55A	109.7	C31—Pd2—S4	99.70 (14)
C56—C55—H55B	109.7	N3—Pd2—S4	178.20 (11)
C54—C55—H55B	109.7	C31—Pd2—S3	174.86 (14)
H55A—C55—H55B	108.2	N3—Pd2—S3	103.49 (11)
C55—C56—C57	111.6 (4)	S4—Pd2—S3	75.20 (4)
