# Supplementary figures and images for: Crystal structure of (piperidine-1-carbo­di­thio­ato-κ2 S,S)[2-(pyridin-2-yl)phenyl-κ2 C 1,N]palladium(II)
Source: Acta Crystallogr E Crystallogr Commun. 2015 Aug 15;71(Pt 9):m166. doi: 10.1107/S2056989015015005 (PMC4555382; doi:10.1107/S2056989015015005)

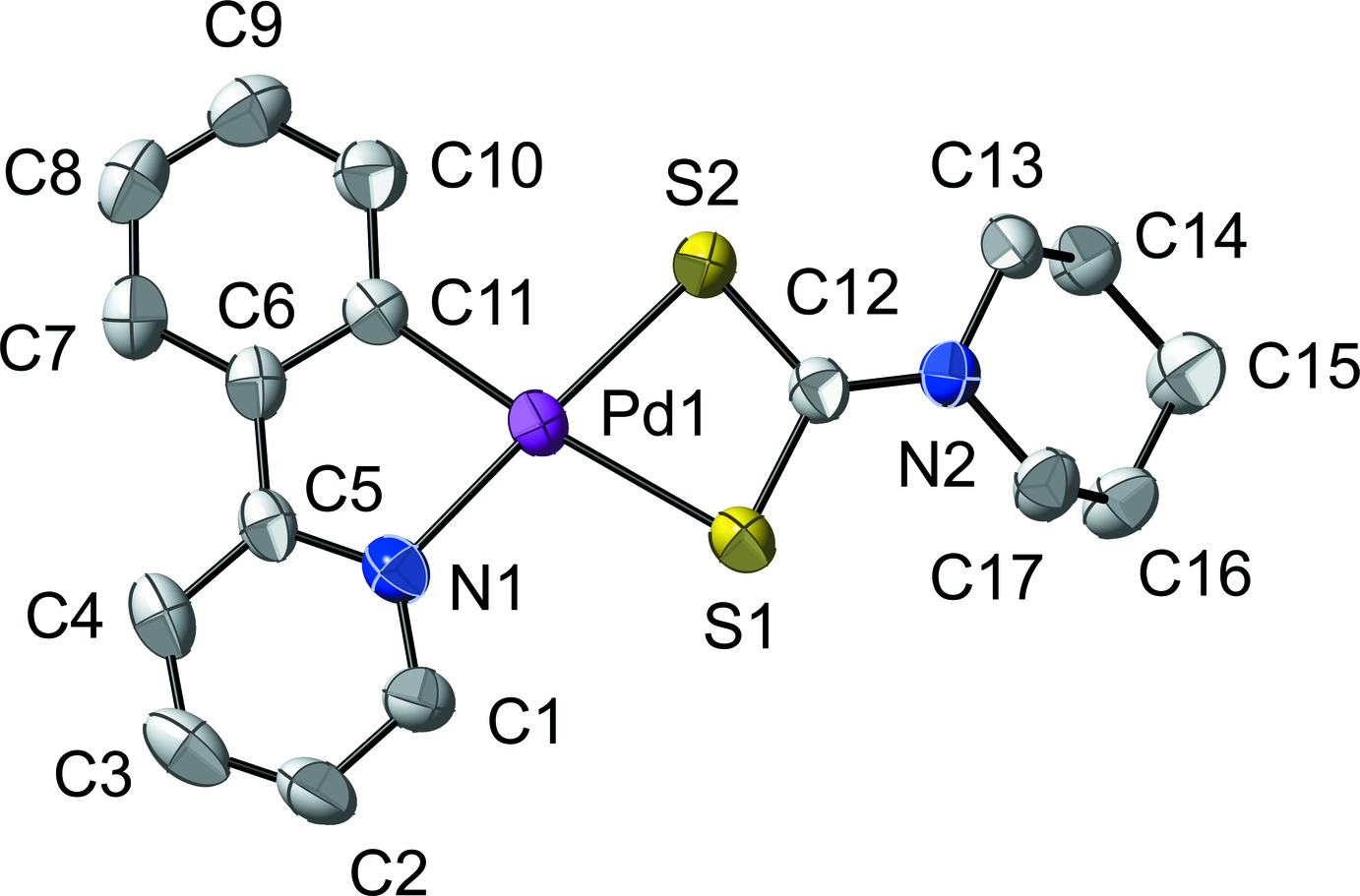

Supplement: Supplementary file 3 [file e-71-0m166-fig1.tif]
